# Difelikefalin improves itch-related sleep disruption in patients undergoing haemodialysis

**DOI:** 10.1093/ndt/gfad245

**Published:** 2023-11-15

**Authors:** Daniel E Weiner, Thilo Schaufler, Kieran McCafferty, Kamyar Kalantar-Zadeh, Michael Germain, Despina Ruessmann, Isabelle Morin, Frédérique Menzaghi, Warren Wen, Sonja Ständer

**Affiliations:** William B. Schwartz MD Division of Nephrology, Tufts Medical Center, Boston, MA, USA; CSL Vifor, Glattbrugg, Switzerland; The Royal London Hospital–Barts Health NHS Trust, London, UK; Division of Nephrology and Hypertension and Kidney Transplantation, University of California, Irvine, Irvine, CA, USA; Baystate Medical Center, Springfield, MA, USA; CSL Vifor, Glattbrugg, Switzerland; CSL Vifor, Meyrin, Switzerland; Cara Therapeutics, Stamford, CT, USA; Cara Therapeutics, Stamford, CT, USA; Center for Chronic Pruritus, Department of Dermatology, University Hospital Münster, Münster, Germany

**Keywords:** chronic kidney disease–associated pruritus, difelikefalin, haemodialysis, itch, sleep

## Abstract

**Background:**

Poor sleep quality is associated with higher mortality and lower quality of life in patients with chronic kidney disease–associated pruritus (CKD-aP). Difelikefalin reduces itch in patients with CKD-aP undergoing haemodialysis (HD). This *post hoc* analysis of the Phase 3 difelikefalin studies (Study 3105 and the pooled dataset from KALM-1 and KALM-2) evaluated whether itch reduction in individuals with CKD-aP improved sleep quality.

**Methods:**

Itch intensity was assessed in patients undergoing HD who had moderate-to-severe CKD-aP treated with intravenous difelikefalin (0.5 µg/kg, three times weekly) (*N* = 222, Study 3105; *N* = 426, KALM-1 and -2) or placebo (*N* = 425, KALM-1 and -2) for 12 weeks, using the Worst Itch Intensity Numerical Rating Scale (WI-NRS). Sleep quality was assessed using the sleep disability question of the 5-D Itch Scale (5-D SDQ) in all studies and, in Study 3105, with the Sleep Quality Numeric Rating Scale (SQ-NRS).

**Results:**

Greater improvements in sleep quality were observed in patients with ≥3-point versus <3-point WI-NRS improvement using SQ-NRS in Study 3105 [mean (95% confidence interval) −5.2 (–5.6, −4.8) vs −1.5 (–2.0, −1.0)] and 5-D SDQ in KALM-1 and -2 [–1.8 (–2.1, −1.6) vs −0.8 (–1.1, −0.4)]. SQ-NRS and WI-NRS scores were highly correlated at both baseline and Week 12 in Study 3105 (Spearman correlation coefficient: 0.77 and 0.84, respectively). Correlations were also observed between 5-D SDQ and WI-NRS scores in Study 3105 and KALM-1 and -2.

**Conclusions:**

In patients undergoing HD with moderate-to-severe CKD-aP, itch reduction with intravenous difelikefalin was associated with improved sleep quality. As disturbed sleep may contribute to mortality and morbidity in CKD-aP, difelikefalin may help to address a major clinical burden by improving sleep quality, secondary to itch relief.

**Trial Registration:**

KALM-1 (NCT03422653), KALM-2 (NCT03636269), Study 3105 (NCT03998163).

Key learning points **What was known:**

- Chronic kidney disease (CKD)-associated pruritus (CKD-aP) affects many patients undergoing haemodialysis (HD).
- Poor sleep quality is associated with higher mortality and lower quality of life in patients with CKD-aP.
- Phase 3 trials in patients with CKD-aP undergoing HD demonstrated clinically meaningful reductions in itch intensity after 12 weeks of treatment with difelikefalin.

**This study adds:**

- This *post hoc* analysis of the Phase 3 difelikefalin studies (Study 3105, KALM-1 and KALM-2) determined that greater improvements in sleep quality were observed in patients with clinically relevant improvements in itch severity.
- Sleep quality and itch severity correlated strongly at baseline and following 12 weeks of treatment with difelikefalin.
- Both measures of sleep quality used in this analysis (the Sleep Quality Numerical Rating Scale questionnaire and the 5-D itch sleep disability question) correlated with itch intensity (assessed through the Worst Itch Intensity Numerical Rating Scale).

**Potential impact:**

- Measurement of sleep quality, as well as itch severity, is important in patients with CKD undergoing dialysis, as this patient population is at increased risk of experiencing CKD-aP.
- As disordered sleep may contribute to mortality and morbidity in CKD-aP, difelikefalin may help to address a major clinical burden by improving sleep quality, secondary to itch relief.

## INTRODUCTION

Chronic kidney disease (CKD)-associated pruritus (CKD-aP) affects many patients undergoing haemodialysis (HD), at least moderately bothering 37% and extremely bothering 7% of patients [1], with evidence of considerable variation in CKD-aP prevalence between countries and institutions, and likely substantial under-recognition [2–5]. Multiple factors have been implicated in the pathophysiology linking kidney disease to pruritus, including dysregulation of the immune system, uraemic toxins and opioid receptor signalling; however, the exact mechanism remains unclear [6, 7].

Despite an uncertain pathophysiology, the impact of CKD-aP on quality of life (QoL) and outcomes such as depression, intravenous antibiotic use, nonadherence with HD sessions and mortality are well evidenced [1, 7]. The role of poor sleep quality as an important contributor to mortality associated with CKD-aP was highlighted in the Dialysis Outcomes and Practice Patterns Study (DOPPS) (1996–2004), in which adjusting for sleep quality attenuated the relationship between pruritus severity and mortality [3]. Furthermore, in a cohort of 170 patients receiving either high-flux HD or online haemodiafiltration, patients with insomnia in addition to pruritus had a lower 20-month survival compared with patients with pruritus alone [8].

The reported number of patients with CKD-aP who also have sleep disturbances is highly variable, with a meta-analysis noting a range of 9%–76% of patients with CKD-aP reporting poor sleep [9]. However, increased risk and prevalence of sleep disruptions in patients with CKD-aP, compared with those without CKD-aP, has been widely reported [3, 9, 10], along with an established relationship between level of itching and extent of sleep disruption [1, 5, 10]. The impact of sleep disruption has been extensively described, with impaired sleep associated with changes in inflammatory status, metabolism and cognition in the short term, and risk of cancer, dementia and metabolic syndrome in the long term [11–13].

As reviewed by Ahdoot, Kalantar-Zadeh and colleagues [14, 15], sleep disturbance forms one aspect of unpleasant ‘symptom clusters’ experienced by patients receiving HD, which can synergistically reduce QoL [16]. Patient- and caregiver-centric initiatives such as the Standardized Outcomes in Nephrology initiative place renewed focus on managing symptoms and QoL in CKD, and describe sleep disturbances and fatigue as among the more debilitating symptoms experienced by patients on HD [16–19]. Although the association between CKD-aP, sleep and QoL is well established, evidence suggests that these symptoms are poorly managed in clinical practice [14, 16].

Difelikefalin is a selective kappa opioid receptor agonist, approved for the treatment of moderate-to-severe CKD-aP in adults undergoing HD [20, 21], which inhibits pruritic and inflammatory signaling [22–25]. Phase 3 studies (the open-label Study 3105 [26] and the pooled results of the randomized placebo-controlled studies KALM-1 [23] and KALM-2 [27]) demonstrated anti-itch efficacy of intravenous 0.5 µg/kg difelikefalin for 12 weeks and favourable safety and tolerability [27].

This paper describes *post hoc* analyses of data from Study 3105 and the pooled KALM-1 and KALM-2 studies, with the aim of determining whether itch reduction improved sleep quality in patients on HD with moderate-to-severe CKD-aP.

## MATERIALS AND METHODS

### Study 3105

Methods and participant disposition for Study 3105 (NCT03998163) have been described previously [26]. Briefly, Study 3105 was a multicentre, open-label, single-arm, Phase 3 study in patients with moderate-to-severe CKD-aP [Worst Itch Intensity Numerical Rating Scale (WI-NRS) ≥5 at baseline] undergoing HD, treated with intravenous difelikefalin (0.5 µg/kg thrice weekly following each HD session) for 12 weeks (*N* = 222).

Itch intensity was evaluated at baseline and at Week 12 using the weekly mean of the 24-hour WI-NRS score, in response to the question ‘Please indicate the intensity of the WORST ITCHING you experienced over the past 24 hours’ with scores ranging from 0 (no itching) to 10 (worst itching imaginable). Complete resolution of pruritus was defined as ≥75% of weekly mean WI-NRS scores equal to 0 or 1 [26]. Patients with weekly mean WI-NRS score at Week 12 of <4 (excluding subjects with complete response), ≥4 to <7, and ≥7, were classified as experiencing mild, moderate and severe pruritus, respectively.

Sleep quality was assessed at the start of the dialysis visit using both the Sleep Quality Numerical Rating Scale (SQ-NRS) questionnaire and the sleep disability question on the 5-D Itch Scale (5-D SDQ). The SQ-NRS asked patients to indicate how much their itch interfered with their sleep over the last 24 hours. Scores ranged from 0 (itch did not interfere with sleep) to 10 (itch completely interfered with sleep), with degree of sleep interference categorized by weekly mean score: <1 (none), ≥1–<4 (mild), ≥4–<7 (moderate) or ≥7 (severe). Complete resolution was defined as all weekly SQ-NRS scores equal to zero [26]. The 5-D SDQ asked patients to ‘Rate the impact of your itching on sleep over the last 2 weeks’, and answers ranged from 1 (never affects sleep) to 5 (delays falling asleep and frequently wakes me up at night) [28].

In this *post hoc* analysis of Study 3105, correlations between WI-NRS and SQ-NRS, and WI-NRS and 5-D SDQ were assessed at baseline and Week 12, and the change from baseline to Week 12 was determined. The correlation between SQ-NRS and 5-D SDQ was assessed for Week 12 data only. All correlations were analysed using Spearman correlation analysis. A paired *t*-test was performed to compare the sleep score at baseline and Week 12 for each WI-NRS improvement category (≥3-point vs <3-point and ≥4-point vs <4-point). All *P*-values were exploratory and should be interpreted descriptively.

### KALM-1 and -2

Detailed methods of the KALM-1 and -2 (NCT03422653/NCT03636269) trials and participant disposition are described elsewhere [23, 27]. Briefly, KALM-1 and -2 were multicentre, placebo-controlled, Phase 3 studies in patients with moderate-to-severe CKD-aP (WI-NRS >4 at baseline in KALM-1, and WI-NRS ≥5 at baseline in KALM-2) undergoing HD, randomized to receive 0.5 µg/kg difelikefalin (*N* = 426) or placebo (*N* = 425) thrice weekly for 12 weeks.

After 12 weeks, patients eligible to receive intravenous difelikefalin 0.5 µg/kg thrice weekly were included in a 52-week open-label extension period [27]. *Post hoc* analyses were performed using data from patients receiving difelikefalin, or combined data from patients receiving difelikefalin or placebo. Itch intensity was evaluated at baseline and at Week 12 by weekly mean of the 24-hour WI-NRS score, and sleep quality was assessed by the 5-D SDQ, as described above. Complete resolution of pruritus was defined as subjects reporting 0 or 1 on at least 80% of the daily WI-NRS scores [27]. Patients with weekly mean WI-NRS score at Week 12 of <4 (excluding subjects with complete response), ≥4 to <7, and ≥7, were classified as experiencing mild, moderate, and severe pruritus, respectively. Change from baseline to Week 12 in WI-NRS or 5-D SDQ scores was calculated and the correlation between WI-NRS and 5-D SDQ was assessed using Spearman's correlation analysis for both Week 12 data and change from baseline to Week 12. A paired t-test was performed to compare 5-D SDQ score between baseline and Week 12 for each WI-NRS improvement category (≥3-point vs <3-point and ≥4-point vs <4-point). All *P*-values were exploratory and should be interpreted descriptively.

## RESULTS

### Baseline demographics

Baseline demographics and clinical characteristics for both KALM-1 and -2 and Study 3105 are shown in Table 1.

**Table 1:** Baseline demographics and clinical characteristics of patients in the pooled KALM-1 and -2 data set and Study 3105.

	Pooled KALM-1 and KALM-2		Study 3105
	Placebo (*n* = 425)	Difelikefalin (*n* = 426)	Difelikefalin (*n* = 222)
Demographics			
Age, mean ± SD, years	58.3 ± 13.5	59.1 ± 12.4	58.1 ± 12.8
Sex, male, *n* (%)	258 (60.7)	249 (58.5)	121 (54.5)
Race, *n* (%)			
Black or African American	114 (26.8)	135 (31.7)	110 (49.5)
White	262 (61.6)	255 (59.9)	96 (43.2)
Other^a^	49 (11.5)	36 (8.5)	16 (7.2)
Region, *n* (%)			
USA	322 (75.8)	335 (78.6)	203 (91.4)
Eastern Europe	60 (14.1)	54 (12.7)	19 (8.6)
Western Europe	31 (7.3)	29 (6.8)	
Asia	12 (2.8)	8 (1.9)	
Clinical characteristics			
Dry body weight at baseline, mean ± SD, kg	82.4 ± 20.6	83.4 ± 20.1	86.6 ± 23.5
Years since diagnosis of ESKD^b^	4.1 (5.3)	3.8 (4.8)	5.9 ± 4.7
Years receiving maintenance HD^b^	3.9 (5.0)	3.5 (4.8)	5.4 ± 4.4
Duration of pruritus, years^b^	2.5 (3.2)	2.1 (3.2)	3.9 ± 3.3
Aetiology of CKD, *n* (%)			
Hypertension	138 (32.5)^c^	122 (28.6)^c^	135 (60.8)
Diabetes	206 (48.5)^c^	225 (52.8)^c^	110 (49.5)
Other	65 (15.3)^c^	61 (14.3)^c^	25 (11.3)
Glomerulonephritis	16 (3.8)^c^	18 (4.2)^c^	11 (5.0)
Calcium, mean ± SD, mg/dL	8.4 ± 0.8^d^	8.8 ± 0.8^d^	8.8 (0.8)
Phosphate, mean ± SD, mg/dL	5.6 ± 2.2^d^	5.6 ± 1.9^d^	5.9 (1.9)
Medication use			
Baseline anti-itch medication use, *n* (%)	163 (38.4)	159 (37.3)	71 (32.0)
Commonly used (>2%) anti-itch medications, *n* (%)			
Diphenhydramine	100 (23.5)	104 (24.4)	49 (22.1)
Hydroxyzine	52 (12.2)	42 (9.9)	14 (6.3)
Gabapentin			3 (1.4)
Hydrocortisone	16 (3.8)	11 (2.6)	
Cetirizine	10 (2.4)	7 (1.6)	
Clemastine	10 (2.4)	7 (1.6)	
Questionnaire scores			
WI-NRS score, mean ± SD	7.2 ± 1.5	7.2 ± 1.4	7.6 ± 1.3
SQ-NRS, mean ± SD			6.6 ± 2.2
5-D itch scale total score, mean ± SD	16.9 ± 3.5	16.8 ± 3.5	17.1 ± 3.5
Skindex-10 scale total score, mean ± SD	36.0 ± 15.1	35.8 ± 14.7	32.9 ± 14.3

Percentages are based on the number of participants in each treatment group.

^a^Race was participant reported; ‘Other’ category includes American Indian or Alaska native, Asian, Native Hawaiian or Pacific Islander, unknown and other.

^b^Values are median (interquartile range) for the KALM-1 and -2 pooled dataset, and mean ± SD for Study 3105.

^c^Diabetes values include patients with diabetes alone; diabetes and hypertension; diabetes, hypertension, and other; or diabetes and other. Hypertension values include patients with hypertension alone or hypertension and other. Glomerulonephritis values include patients with glomerulonephritis alone or glomerulonephritis and other.

^d^Conversion factors for units were as follows: for calcium, mg/dL to mmol/L, ×0.2495; and for phosphate, mg/dL to mmol/L, ×0.3229.

ESKD, end-stand kidney disease; SD, standard deviation.

### Study 3105

**Figure 1:**
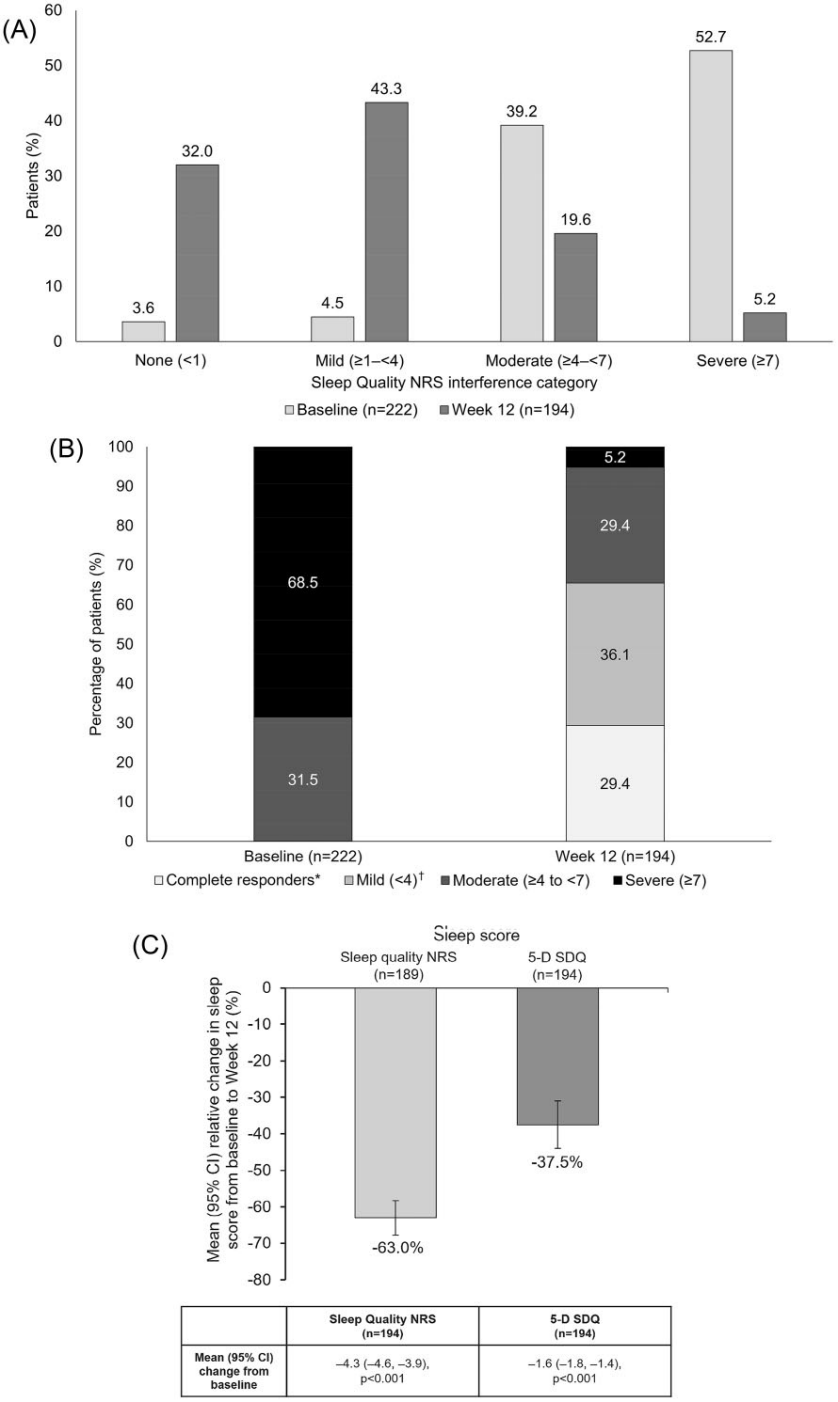
Sleep disruption and itch severity in Study 3105 patients receiving difelikefalin, at baseline and Week 12. (**A**) The proportion of patients with no, mild, moderate and severe sleep disruption as measured by the Sleep Quality NRS score. (**B**) The proportion of patients with complete response and mild, moderate or severe pruritus as measured by the WI-NRS score. (**C**) Change in Sleep Quality NRS and 5-D SDQ scores. *Complete responder defined as ≥75% of weekly mean WI-NRS scores equal to 0 or 1. ^†^Mild (≥0 to <4) data excludes patients with complete response. 5-D SDQ, 5-D Itch Scale sleep disability question; NRS, Numeric Rating Scale; WI-NRS, Worst Itch Intensity Numeric Rating Scale.

The proportion of patients with itch-related sleep disruption at baseline and following 12 weeks of difelikefalin treatment is shown in Fig. 1A. Answers to the SQ-NRS questionnaire at baseline revealed 3.6%, 4.5%, and 39.2% of patients reported no, mild, or moderate sleep disruption, respectively, while 52.7% of patients reported severe itch-related sleep disruption. In addition, 13.5% of patients reported an SQ-NRS score ≥9. For WI-NRS, 31.5% reported moderate pruritus (score ≥4 to <7) and 68.5% reported severe pruritus (score ≥7) at baseline (Fig. 1B).

At Week 12, 32.0%, 43.3% and 19.6% of patients reported no, mild or moderate sleep disruption, respectively. Only 5.2% of patients reported severe itch-related sleep disruption, while no patients scored ≥9 on the SQ-NRS (Fig. 1A). At Week 12, 29.4% of patients receiving difelikefalin had a complete response, 36.1% reported mild pruritus (WI-NRS score <4, excluding complete responders), 29.4% reported moderate pruritus (WI-NRS score ≥4 to <7) and 5.2% reported severe pruritus (WI-NRS score ≥7).

Changes in SQ-NRS score equated to a significant mean change [95% confidence interval (CI)] from baseline to Week 12 of −4.3 (–4.6, −3.9) (*P* < .001), and a significant relative percentage improvement in SQ-NRS score from baseline to Week 12 [mean (95% CI) −63.0% (–67.8, −58.3), *P* < .001] (Fig. 1C). Significant absolute [–1.6 (–1.8, −1.4), *P* < .001] and relative [–37.5% (–44.0, −31.0), *P* < .001] improvements were also observed in 5-D SDQ from baseline to Week 12 (Fig. 1C).

**Figure 2:**
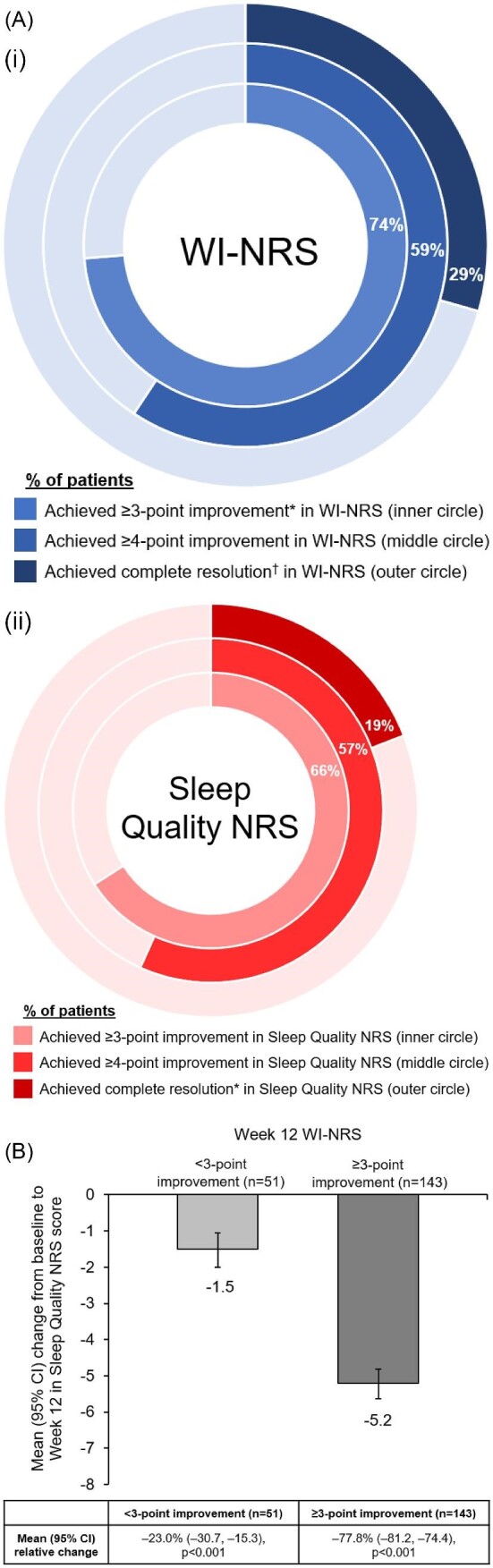

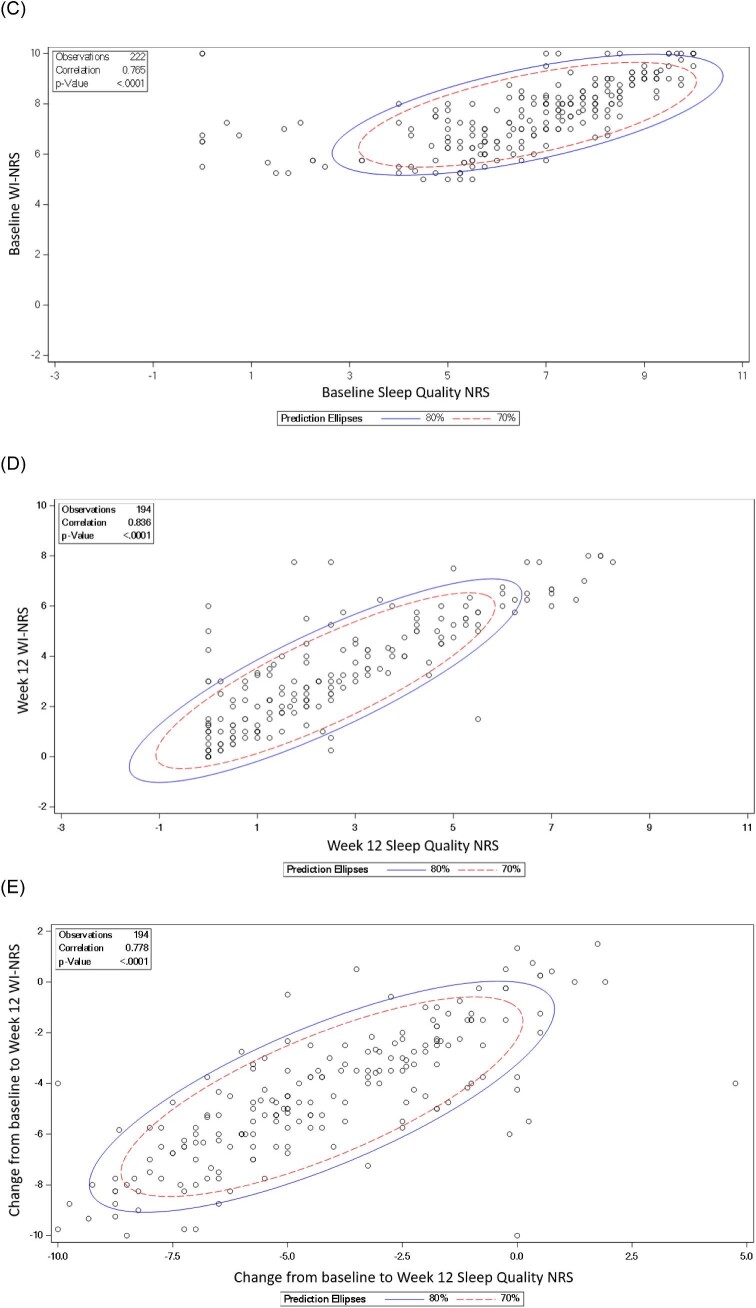
Relationship between WI-NRS and Sleep Quality NRS scores in Study 3105 patients receiving difelikefalin. (**A**) The proportion of patients with ≥3-point improvement, ≥4-point improvement or complete response for WI-NRS (i) and Sleep Quality NRS scores (ii) following 12 weeks receiving difelikefalin. (**B**) Change in Sleep Quality NRS score from baseline in patients with <3-point and ≥3-point improvement in WI-NRS score following 12 weeks receiving difelikefalin. (**C**) Correlation between baseline WI-NRS and Sleep Quality NRS scores. (**D**) Correlation between Week 12 WI-NRS and Sleep Quality NRS scores. (**E**) Correlation between change from baseline to Week 12 WI-NRS and Sleep Quality NRS scores. (Ai) *A threshold reduction of 3 points or more in itch intensity was defined as clinically meaningful for this patient population [31]. ^†^Complete resolution indicates ≥75% of weekly mean WI-NRS scores 0 or 1. *n*/*N* were 143/194 for ≥3-point improvement, 115/194 for ≥4-point improvement and 57/194 for complete resolution. (Aii) *Complete resolution indicates all Sleep Quality NRS scores equal to 0. *n*/*N* were 128/194 for ≥3-point improvement, 110/194 for ≥4-point improvement and 37/194 for complete resolution.

At Week 12, the majority of patients achieved ≥3-point or ≥4-point improvement in WI-NRS (73.7% and 59.3%, respectively) and SQ-NRS scores (66.0% and 56.7%, respectively). Complete resolution of WI-NRS (0 or 1 on ≥75% of daily WI-NRS scores) and SQ-NRS (0 for all SQ-NRS scores) scores was observed in 29.4% and 19.1% of patients, respectively (Fig. 2A). Figure 2B shows the summary of change from baseline to Week 12 in SQ-NRS score, by WI-NRS score improvement categories from baseline to Week 12 (≥3-point, <3-point). In patients with ≥3-point improvement in WI-NRS, the mean change (95% CI) in SQ-NRS score from baseline to Week 12 was −5.2 (95% CI −5.6, −4.8) (*P* < .001), whereas in patients with a <3-point improvement in WI-NRS, change in sleep score was −1.5 (95% CI −2.0, −1.0) (*P* < .001). This equated to a relative percentage improvement in SQ-NRS score from baseline to Week 12 of −77.8% (95% CI −81.2, −74.4) (*P* < .001) for patients with ≥3-point improvement in WI-NRS, and −23.0% (95% CI −30.7, −15.3) (*P* < .001) for patients with a <3-point improvement in WI-NRS. Similar results were observed in SQ-NRS scores from patients with ≥4-point versus <4-point WI-NRS improvement (Supplementary data, Fig. S1). Strong correlations were observed between the weekly average SQ-NRS score and the weekly average WI-NRS at baseline [Spearman correlation coefficient (95% CI) 0.77 (0.70, 0.81), *P* < .0001] (Fig. 2C) as well as at Week 12 [Spearman correlation coefficient (95% CI) 0.84 (0.79, 0.87), *P* < .0001] (Fig. 2D) and changes from baseline to Week 12 [Spearman correlation coefficient (95% CI) 0.78 (0.72, 0.83), *P* < .0001] (Fig. 2E).

**Figure 3:**
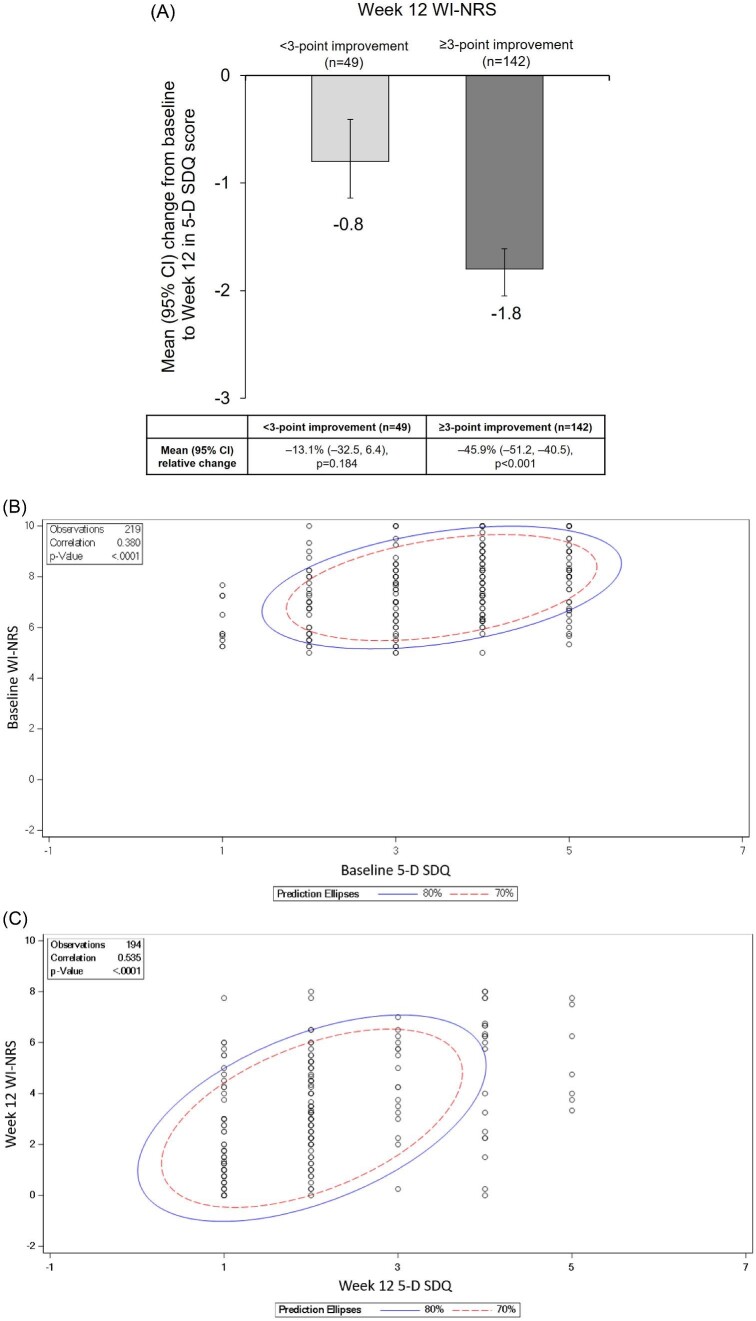

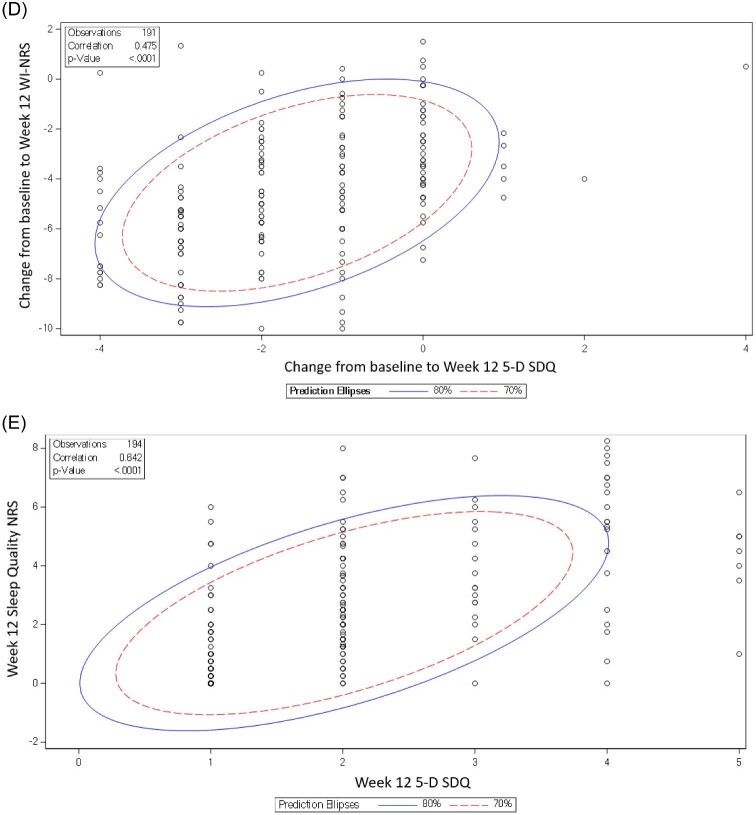
Relationship between WI-NRS and 5-D SDQ scores at baseline and at Week 12 in Study 3105. (**A**) Change in 5-D SDQ score from baseline in Study 3105 patients with <3-point and ≥3-point improvement in WI-NRS score following 12 weeks receiving difelikefalin. (**B**) Correlation between baseline WI-NRS and 5-D SDQ scores in Study 3105 patients. (**C**) Correlation between Week 12 WI-NRS and 5-D SDQ scores in Study 3105 patients. (**D**) Correlation between change from baseline to Week 12 WI-NRS and 5-D SDQ scores in Study 3105 patients. (**E**) Correlation between Week 12 Sleep Quality NRS scores and 5-D SDQ scores in Study 3105 patients. Safety population, *n* = 222. 5-D SDQ, 5-D Itch Scale sleep disability question; WI-NRS, Worst Itch Intensity Numeric Rating Scale.

Figure 3A shows the mean change (95% CI) from baseline to Week 12 for 5-D SDQ score by WI-NRS score improvement from baseline to Week 12 (≥3-point vs <3-point). Patients with a ≥3-point improvement versus <3-point improvement in WI-NRS had a −1.8 (95% CI −2.1, −1.6) (*P* < .001) change compared with a −0.8 (95% CI −1.1, −0.4) (*P* < .001) change in 5-D SDQ score from baseline, following 12 weeks’ difelikefalin treatment. This equated to a relative percentage improvement in 5-D SDQ score from baseline to Week 12 of −45.9% (–51.2, −40.5) (*P* < .001), for patients with ≥3-point improvement in WI-NRS, and −13.1% (–32.5, 6.4) (*P* = .184), for patients with a <3-point improvement in WI-NRS. Similar results were observed with 5-D SDQ scores from patients with ≥4-point versus <4-point WI-NRS improvements (Supplementary data, Fig. S2). A moderate correlation was observed between the 5-D SDQ score and WI-NRS at baseline [Spearman correlation coefficient (95% CI) 0.38 (0.26, 0.49), *P* < .0001], while moderate correlations were observed at Week 12 [Spearman correlation coefficient (95% CI) 0.54 (0.43, 0.63), *P* < .0001], and for the changes from baseline to Week 12 [Spearman correlation coefficient (95% CI) 0.47 (0.36, 0.58), *P* < .0001] (Fig. 3B–D). A moderate correlation was also observed between SQ-NRS score and 5-D SDQ score at Week 12: 0.64 (95% CI 0.55, 0.72) (*P* < .0001) (Fig. 3E).

### KALM-1 and -2

At baseline, 9.2% and 10.3% of KALM-1 and -2 patients randomized to receive placebo or difelikefalin, respectively, reported that itching ‘never affected sleep’; 18.4% and 19.1%, respectively, reported occasional delays in falling asleep; and 23.3% and 24.6%, respectively, reported frequent delays in falling asleep due to itch. Delays falling asleep coupled with occasional night waking was reported by 28.9% of patients receiving placebo and 29.2% of patients receiving difelikefalin, whereas delays falling asleep and frequently being woken at night was reported by 20.2% of patients receiving placebo and 16.7% of patients receiving difelikefalin. At baseline, 45.4% and 43.4% of patients in the placebo and difelikefalin groups reported moderate pruritus (WI-NRS score ≥4 to <7), respectively, and 54.6% and 56.6% reported severe pruritus (WI-NRS score ≥7).

**Figure 4:**
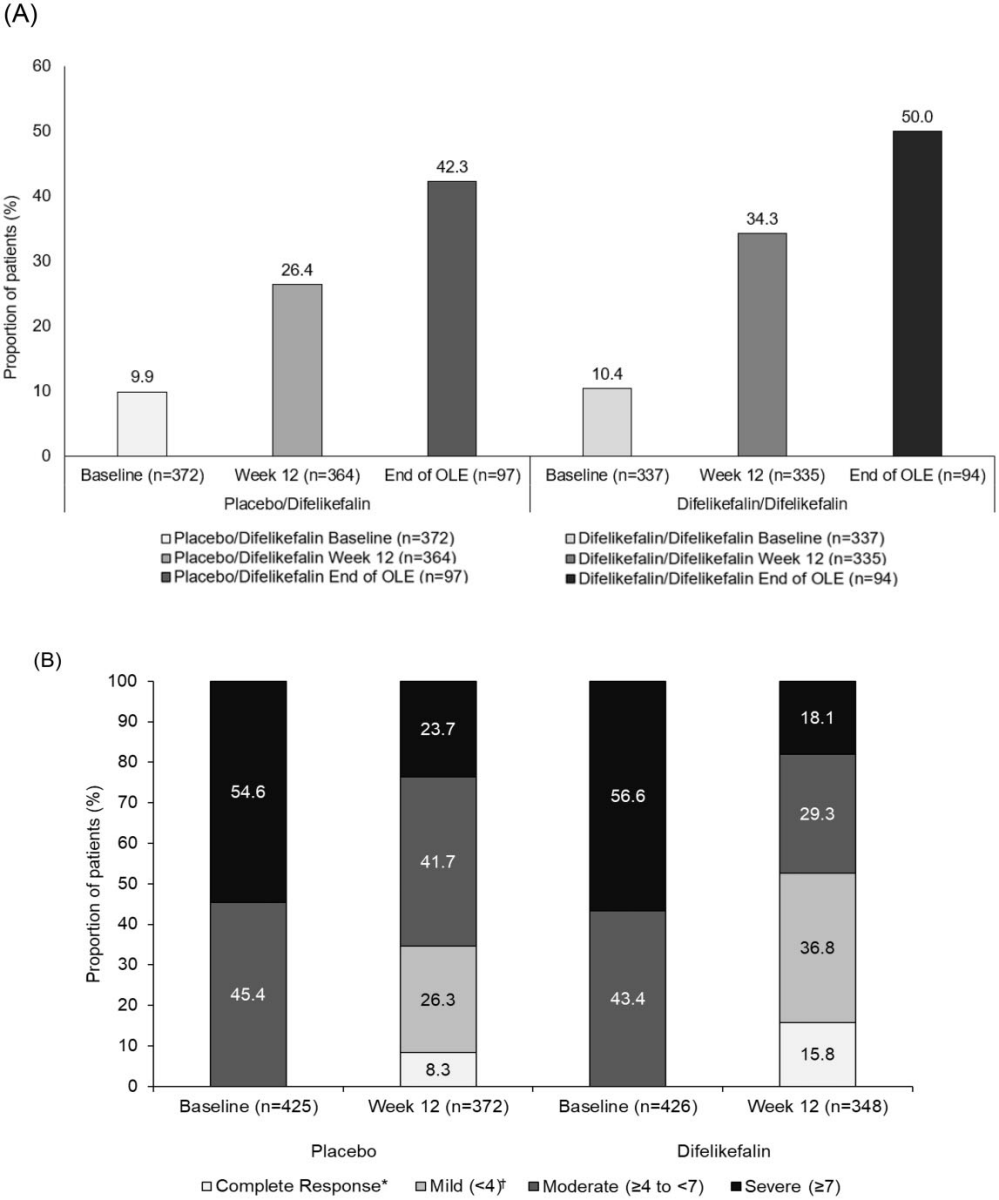
5-D SDQ scores and itch severity in KALM-1 and -2 patients receiving either placebo or difelikefalin. (**A**) The proportion of KALM-1 and -2 patients receiving either placebo/difelikefalin or difelikefalin/difelikefalin reporting ‘never affects sleep’ on the 5-D SDQ score, at baseline, Week 12 and the end of the open label extension (OLE) period. (**B**) The proportion of KALM-1 and -2 patients receiving either placebo or difelikefalin reporting complete response, mild, moderate or severe pruritus, as measured by WI-NRS, at baseline and Week 12. Complete response defined as patients where 80% or more of their weekly values were 0 or 1. ^†^Mild (≥0 to <4) data exclude subjects with complete response.

At baseline, approximately 10% of patients in either the placebo or difelikefalin groups reported that itching ‘never affects sleep’ on the 5-D SDQ score. Following 12 weeks and 64 weeks of receiving difelikefalin, itching never affecting sleep was reported by 34.3% and 50.0% of patients, respectively, compared with 26.4% of patients on placebo at Week 12. For patients on placebo who subsequently received difelikefalin in the open label extension period, 42.3% reported that itch never affected sleep after 52 weeks (Fig. 4A).

With regard to itch severity, 8.3% of patients receiving placebo had a complete response, 26.3% reported mild pruritus (WI-NRS score <4, excluding complete responders), 41.7% reported moderate pruritus (WI-NRS score ≥4 to <7) and 23.7% reported severe pruritus (WI-NRS score ≥7) at Week 12 (Fig. 4B). Whereas for patients receiving difelikefalin, 15.8% of patients reported a complete response, 36.8% reported mild pruritus (WI-NRS score <4, excluding complete responders), 29.3% reported moderate pruritus (WI-NRS score ≥4 to <7) and 18.1% reported severe pruritus (WI-NRS score ≥7) at Week 12 (Fig. 4B).

**Figure 5:**
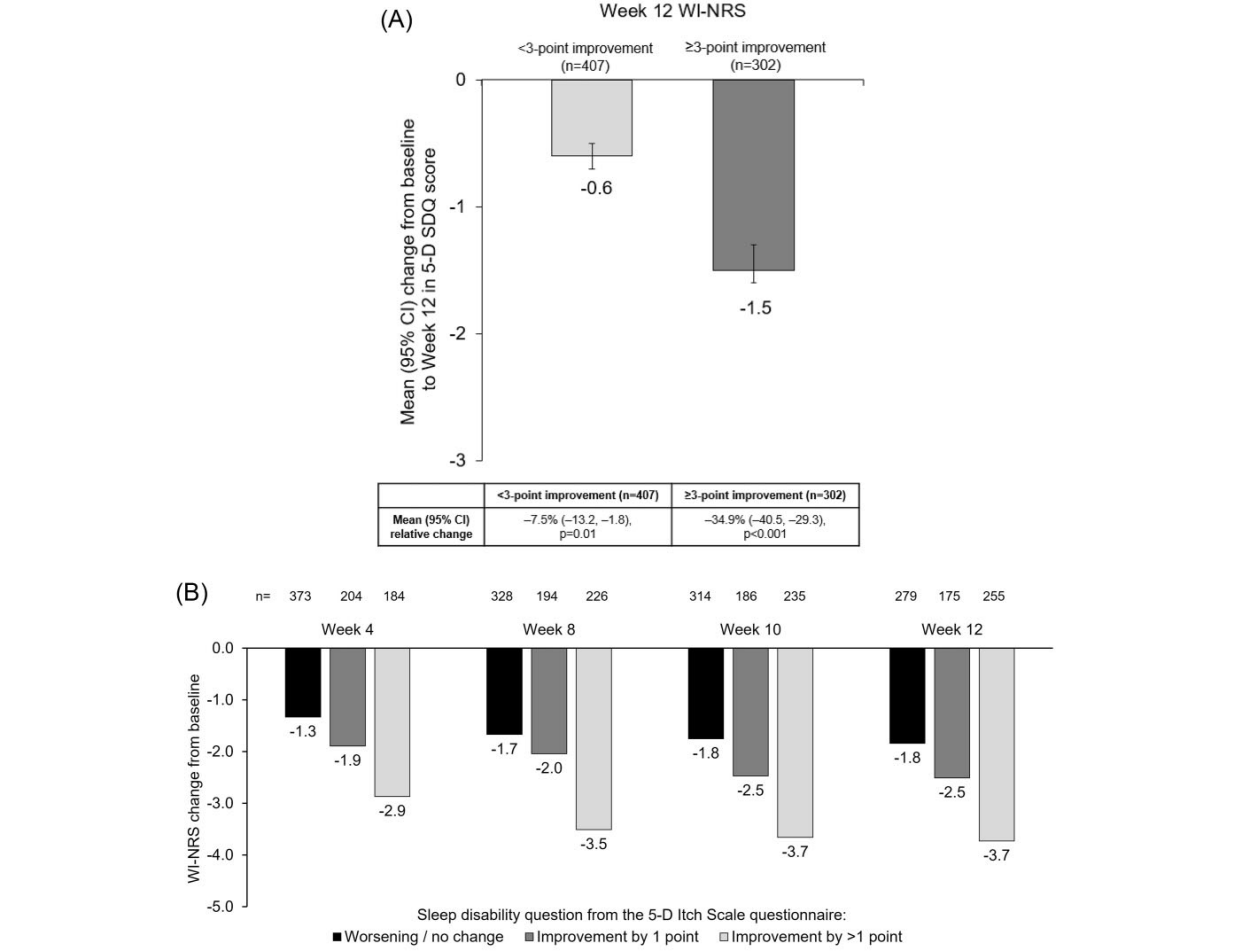

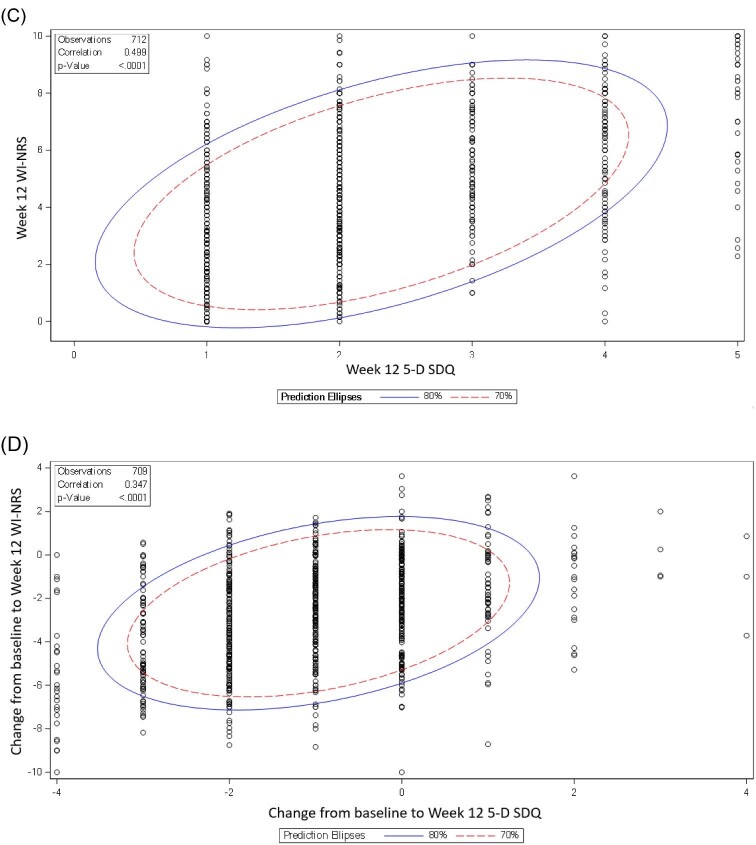
Relationship between WI-NRS and 5-D SDQ scores in KALM-1 and -2 patients receiving placebo or difelikefalin. (**A**) Change in 5-D SDQ score from baseline to Week 12 by improvement in WI-NRS score (<3-point and ≥3-point) at Week 12 in patients receiving difelikefalin or placebo. (**B**) Change from baseline WI-NRS score by improvement in the 5-D SDQ score at Weeks 4, 8, 10 and 12 in patients receiving difelikefalin or placebo. (**C**) Correlation between Week 12 WI-NRS and 5-D SDQ scores in patients receiving difelikefalin or placebo. (**D**) Correlation between change from baseline to Week 12 WI-NRS and 5-D SDQ scores in patients receiving difelikefalin or placebo. Intention-to-treat population, *n* = 851. 5-D SDQ, 5-D Itch Scale sleep disability question; WI-NRS, Worst Itch Intensity Numeric Rating Scale.

For further KALM-1 and -2 analysis, data were combined from patients receiving either difelikefalin or placebo for 12 weeks. A greater improvement in 5-D SDQ score was observed in patients with ≥3-point improvement in WI-NRS score, compared with patients with <3-point improvement in WI-NRS [mean (95% CI) −1.5 (–1.6, −1.3), *P* < .001 vs −0.6 (–0.7, −0.5), *P* < .001] (Fig. 5A). This equated to a relative improvement of −34.9% (–40.5, −29.3) (*P* < .001) in patients with ≥3-point improvement in WI-NRS score, and −7.5% (–13.2, −1.8) (*P* = .01) in patients with <3-point improvement. Similarly, patients with a ≥3-point improvement in WI-NRS score were more likely to have a >1-point improvement in 5-D SDQ score over time (Fig. 5B). Similar results were observed for 5-D SDQ scores in patients with ≥4-point versus <4-point WI-NRS improvements (Supplementary data, Fig. S3). Spearman correlation coefficients to determine the correlation between WI-NRS score and the 5-D SDQ score for Week 12, and change from baseline at Week 12, were 0.50 (95% CI 0.44, 0.55) and 0.35 (95% CI 0.28, 0.41), respectively (Fig. 5C and D).

## DISCUSSION

In *post hoc* analyses of Study 3105 and the KALM-1 and -2 Phase 3 trials of difelikefalin, itch reduction was associated with improved sleep quality among HD patients with moderate-to-severe CKD-aP.

Baseline data presented in this paper highlight the substantial impact of CKD-aP on sleep quality, with more than half of patients reporting severe itch-related sleep disruption via the SQ-NRS questionnaire in Study 3105, and almost half of patients reporting delays in falling asleep and being woken either occasionally or frequently at night via the 5-D SDQ in KALM-1 and -2. These findings are consistent with previous studies reporting substantial sleep problems in dialysis patients with CKD-aP [5, 8]. For example, You *et al.* described lack of energy and trouble falling asleep, trouble falling asleep and trouble staying asleep, and feeling tired/lack of energy and trouble falling asleep as the most prevalent pairings of symptoms experienced in a cohort of patients on HD [19].

The current *post hoc* analysis of Study 3105 found that patients reaching a clinically meaningful change in pruritus of ≥3 points on WI-NRS with difelikefalin had a greater improvement in sleep quality compared with patients reporting <3-point improvement [29]. Similar results were observed in patients reporting ≥4-point and <4-point WI-NRS improvement. Further analyses from Study 3105 revealed a moderate-to-strong or strong correlation between a reduction in WI-NRS score and an improvement in SQ-NRS score, depending on the timepoint studied, while weak-to-moderate or moderate correlations were observed between WI-NRS and 5-D SDQ scores. Importantly, a correlation was noted between SQ-NRS and 5-D SDQ scores, indicating that both SQ-NRS and 5-D SDQ provide clinically relevant measures of sleep quality in patients with CKD-aP, and allowing for an analysis of the impact of itch reduction on sleep quality in the KALM-1 and -2 studies, which reported 5-D itch data, but not SQ-NRS.

In KALM-1 and -2, more than a third of patients receiving difelikefalin reported that itch never affected sleep at Week 12, compared with a quarter of patients receiving placebo. Following open-label treatment with difelikefalin from Week 12 to Week 64, approximately half of patients reported that itch never affected sleep, suggesting that improvements in sleep may continue to increase with extended treatment.

A greater improvement in 5-D SDQ score for patients reaching a clinically meaningful change via WI-NRS, and a correlation between WI-NRS and 5-D SDQ scores, were also observed in KALM-1 and -2. Because these data were gathered from patients receiving either difelikefalin or placebo, this correlation demonstrates that the improvement in sleep observed in KALM-1 and -2 is due to reduction in itch, rather than any direct effects of difelikefalin on sleep or central nervous system activity.

Overall, analysis of these three Phase 3 studies demonstrates a moderate-to-strong correlation between reduced itch intensity and improved sleep quality in patients with moderate-to-severe CKD-aP on HD. As these *post hoc* analyses demonstrate an improvement in sleep with itch reduction, the significant reduction in itch observed with difelikefalin in trials could lead to meaningful improvements in sleep in patients with CKD-aP. This is consistent with a previous Phase 2 study by Fishbane and colleagues that reported significantly reduced itch alongside significantly improved sleep in patients receiving 0.5 µg/kg difelikefalin thrice weekly for 8 weeks, compared with placebo [30]. The authors also reported a significant improvement in the score for the entire 5-D itch disability domain with difelikefalin versus placebo [30]. Furthermore, a relationship between itching and the extent of sleep disruption has been described elsewhere [5, 10, 30]. In a study by Narita and colleagues, patients on HD with severe pruritus, as measured by the visual analogue scale, were significantly more likely to experience issues relating to sleep, with 72% experiencing sleep disturbances, 25% waking more than a few times a night and 16% experiencing sleeplessness [10].

A limitation of these *post hoc* analyses from Study 3105 and KALM-1 and -2 is the differences in trial design, such as placebo control, and methodologies, such as sleep scales. The *post hoc* exploratory nature of this analysis requires the findings and statistical significance to be interpreted with caution.

## CONCLUSION

Among patients undergoing HD with moderate-to-severe CKD-aP, itch reduction correlated with improved sleep quality. Reduction of itch with difelikefalin, as demonstrated in randomized controlled and open-label trials, leads to sleep benefit for patients. Because poor sleep is a major contributor to physical and mental morbidity, as well as the enhanced mortality in patients with CKD-aP, difelikefalin may help to address a major clinical burden and improve the day-to-day life for patients on HD by improving sleep quality, secondary to itch relief.

## Supplementary Material

gfad245_Supplemental_File

## Data Availability

The data underlying this article will be shared on reasonable request to the corresponding author.
