# Supplementary material for: Difelikefalin improves itch-related sleep disruption in patients undergoing haemodialysis
Source: Nephrol Dial Transplant. 2023 Nov 15;39(7):1125–37. doi: 10.1093/ndt/gfad245 (PMC11210984; doi:10.1093/ndt/gfad245)
Supplement: gfad245_Supplemental_File [file gfad245_supplemental_file.docx]

**Supplementary figure 1:** Change in Sleep Quality NRS score from baseline to Week 12 in Study 3105 patients with <4-point and ≥4-point improvement in WI-NRS score following 12 weeks receiving difelikefalin.


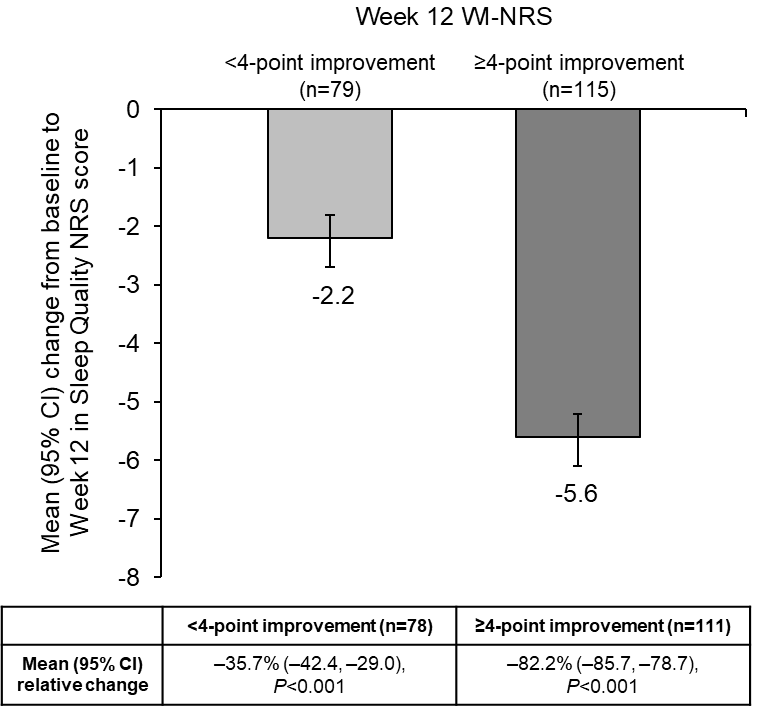


CI, confidence interval; NRS, Numerical Rating Scale; WI-NRS, Worst Itching Intensity Numerical Rating Scale.

**Supplementary figure 2:** Change in 5-D SDQ score from baseline to Week 12 in Study 3105 patients with <4-point and ≥4-point improvement in WI-NRS score following 12 weeks receiving difelikefalin.


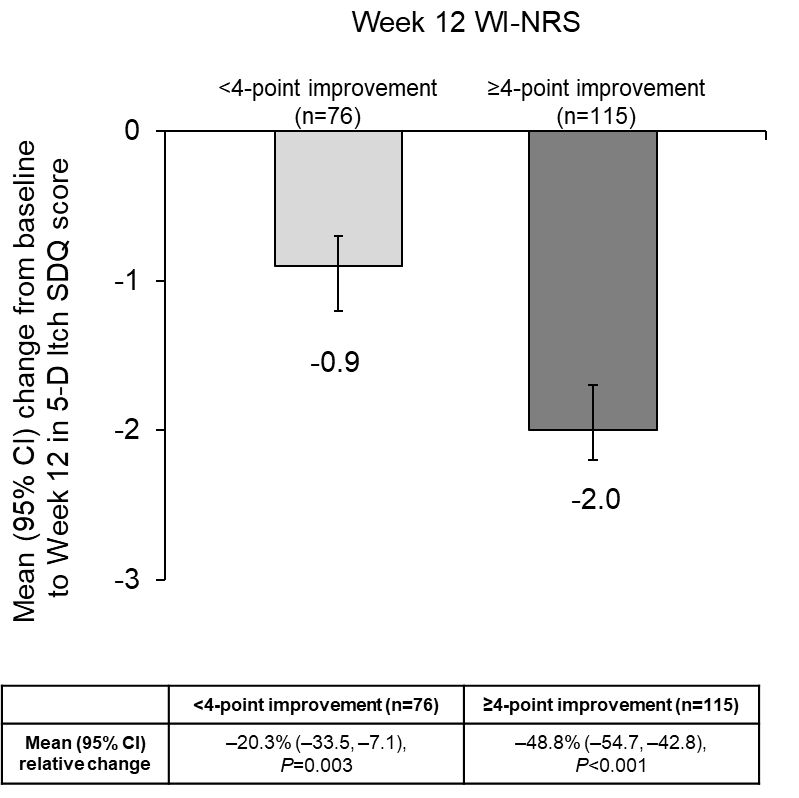


CI, confidence interval; SDQ, sleep disability question; WI-NRS, Worst Itching Intensity Numerical Rating Scale.

**Supplementary figure 3:** Change in 5-D SDQ score from baseline to Week 12 in KALM-1 and ‌‑2 patients with <4-point and ≥4-point improvement in WI-NRS score following 12 weeks receiving difelikefalin or placebo.


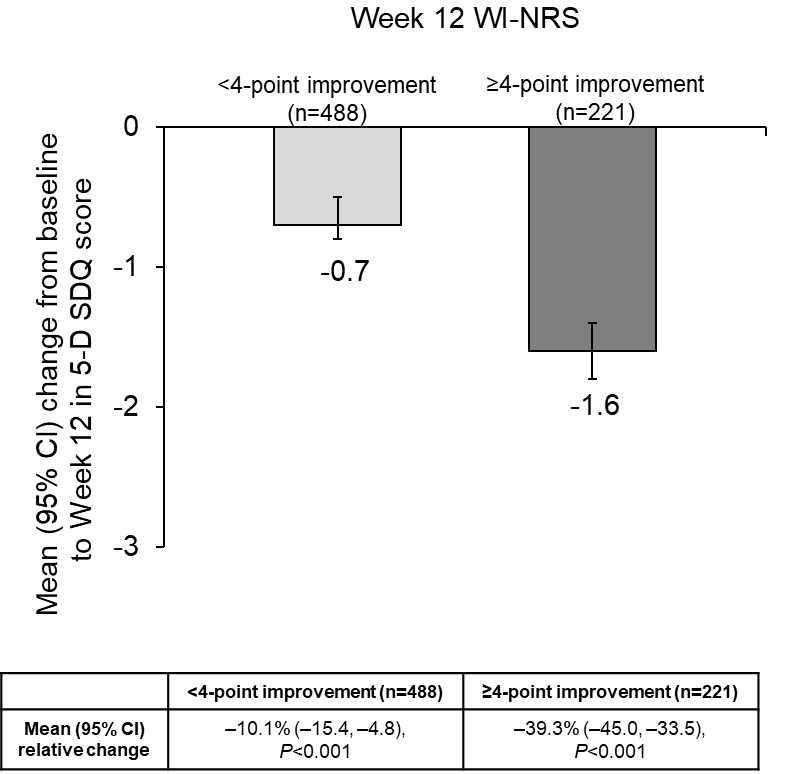


CI, confidence interval; SDQ, sleep disability question; WI-NRS, Worst Itching Intensity Numerical Rating Scale.
